# CsPbBr_3_ Nanocrystals as Bottom Interface Nucleation Seeds for Printing Oriented FAPbI_3_ Thin Films: An In Situ Study

**DOI:** 10.1002/smll.202505895

**Published:** 2025-08-20

**Authors:** Altantulga Buyan‐Arivjikh, Jascha Fricker, Thomas Baier, Xiaojing Ci, Lixing Li, Deepika Gaur, Lakshminarayana Polavarapu, Matthias Schwartzkopf, Sarathlal Koyilot Vayalil, Peter Müller‐Buschbaum

**Affiliations:** ^1^ Chair for Functional Materials Department of Physics TUM School of Natural Sciences Technical University of Munich James‐Franck‐Straße 1 85748 Garching Germany; ^2^ Department of Physical Chemistry CINBIO Universidade de Vigo Materials Chemistry and Physics Group Campus Universitario As Lagoas Marcosende Vigo 36310 Spain; ^3^ Deutsches Elektronen‐Synchrotron DESY Notkestraße 85 22607 Hamburg Germany; ^4^ Department of Physics, Applied Science Cluster UPES Dehradun 248007 India

**Keywords:** crystallization, in situ study, perovskite film, printing, seed crystals

## Abstract

The exceptional optoelectronic properties of lead halide perovskites are highly sensitive to processing conditions, as uncontrolled crystallization driven by random nucleation often results in defect‐rich active layers that impair device performance. Achieving controlled and oriented crystallization in printed films remains a major challenge. To address this, we introduce a pre‐deposited CsPbBr_3_ nanocrystal seed layer at the bottom interface to guide crystallization and suppress defect formation. This strategy is evaluated via an in situ study on FAPbI_3_, offering mechanistic insights into the influence of seeding on film growth and optoelectronic quality. Using in situ grazing‐incidence wide‐angle X‐ray scattering, transmission‐mode UV–vis absorption, and photoluminescence spectroscopy, phase evolution and seed‐mediated growth kinetics are tracked. Seeding accelerates the transition from the photoinactive δ‐phase to the photoactive α‐phase, yielding a crystallization rate constant over six times higher than in unseeded films. Moreover, the seed layer governs the crystallographic orientation of the resulting perovskite film, leading to improved optical absorption and reduced defect density.

## Graphical abstract

A bottom‐interface seeding approach with a pre‐templated CsPbBr_3_ nanocrystal layer is used to regulate the crystallization process and suppress defects in the printing of FAPbI_3_ layers. An earlier and faster transition from the photoinactive δ‐phase to the photoactive α‐phase is found, with a crystallographic orientation of the bulk perovskite film dictated by the seed crystals.

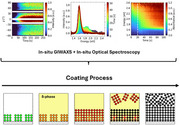

## 1 Introduction

Lead halide perovskites have gained significant research attention over the past decade owing to their favorable optoelectronic properties and exceptional performance.^[^ ^1^, ^2^, ^3^ ^]^ Unlike conventional semiconductors, this class of materials can crystallize out of solution at relatively low temperatures, opening the door for solution‐processable, cost‐effective, and highly efficient optoelectronic devices for applications in fields such as photovoltaics,^[^ ^4^ ^]^ photodetection,^[^ ^5^, ^6^ ^]^ light emission^[^ ^7^, ^8^ ^]^ and beyond. In lead halide perovskites, FAPbI_3_ (FAPI) stands out as particularly compelling due to its suitability for photovoltaic applications, owing to its ideal bandgap relative to MAPbI_3_ and CsPbI_3_.^[^ ^9^, ^10^ ^]^ Most devices are based on polycrystalline thin films for which thin film crystallinity and morphology are key factors affecting the perovskite properties. Therefore, controlling the crystallization dynamics of lead halide perovskites is essential for optimizing film quality and device performance.

Various strategies have been explored, including solvent engineering,^[^ ^11^, ^12^ ^]^ additive incorporation,^[^ ^13^, ^14^, ^15^ ^]^ interface modification,^[^ ^16^, ^17^ ^]^ post synthesis treatment,^[^ ^18^, ^19^ ^]^ etc. Each aiming to regulate nucleation and growth. Among these, the use of seed crystals has emerged as a promising route to bypass spontaneous nucleation and guide crystallographic orientation in thin films.^[^ ^20^, ^21^, ^22^ ^]^ To this end, diverse seeding strategies have been developed, such as incorporating seeds into the precursor solution,^[^ ^23^ ^]^ embedding them into a PbI_2_ layer during two‐step deposition,^[^ ^24^, ^25^ ^]^ or depositing seeds on the top interfaces of the active layer.^[^ ^26^, ^27^ ^]^ These approaches have proven effective in enhancing crystallinity, increasing grain size, and reducing trap densities. However, they also present distinct limitations: solution‐based seeding suffers from limited shelf life and colloidal instability due to the solubility of halide perovskites in polar solvents^[^ ^28^, ^29^ ^]^ in systems that are already unstable and susceptible to deterioration, embedding seeds into intermediate layers increases process complexity,^[^ ^30^ ^]^ and top‐layer seeding fails to address defect formation at the buried substrate interface.^[^ ^31^ ^]^ These factors pose challenges to scalable, high‐throughput manufacturing. Addressing these challenges requires seed materials that are cost‐effective, relatively stable, lattice‐matched to the target perovskite phase,^[^ ^31^ ^]^ and compatible with bottom‐interface integration.^[^ ^32^ ^]^ Low‐cost lead halide perovskite‐based seeds can fulfill these criteria, with their structural similarity facilitating epitaxial growth.^[^ ^20^, ^21^ ^]^ In particular, inorganic variants such as CsPbBr_3_ exhibit greater environmental and colloidal stability than organic counterparts,^[^ ^9^, ^33^ ^]^ making them well‐suited for use in robust bottom‐seeding strategies. In this work, we introduce a bottom‐interface seeding approach using CsPbBr_3_ nanocrystals (NCs) as nucleation centers for the fabrication of printed FAPI thin films. The method involves depositing a NC layer at the substrate interface, followed by a brief surface treatment prior to ink deposition. This approach promotes bottom‐up crystallization from the buried interface while maintaining compatibility with scalable processing workflows.

To enable scalability and industry‐compatible processing,^[^ ^34^, ^35^ ^]^ we select slot‐die coating as a deposition technique and conduct a multimodal in situ study via grazing incidence wide‐angle X‐ray scattering (GIWAXS), transmission mode UV–vis absorption (UV–vis), and photoluminescence (PL) spectroscopy for exploring the film formation kinetics during the deposition process. Our results showcase a highly dynamic film formation process with a competitive growth between isotropically oriented grains originating from random nucleation events and (001) oriented crystallites dictated by the pre‐templated orientation of the CsPbBr_3_ NC seeds. Due to seeding, the resulting thin films exhibit a superior absorbance and a reduced defect density compared to their unseeded counterparts.

## 2 Results and Discussion

### 2.1 CsPbBr_3_ Nanocrystal Seeds and Templated Films

**Figure 1:**
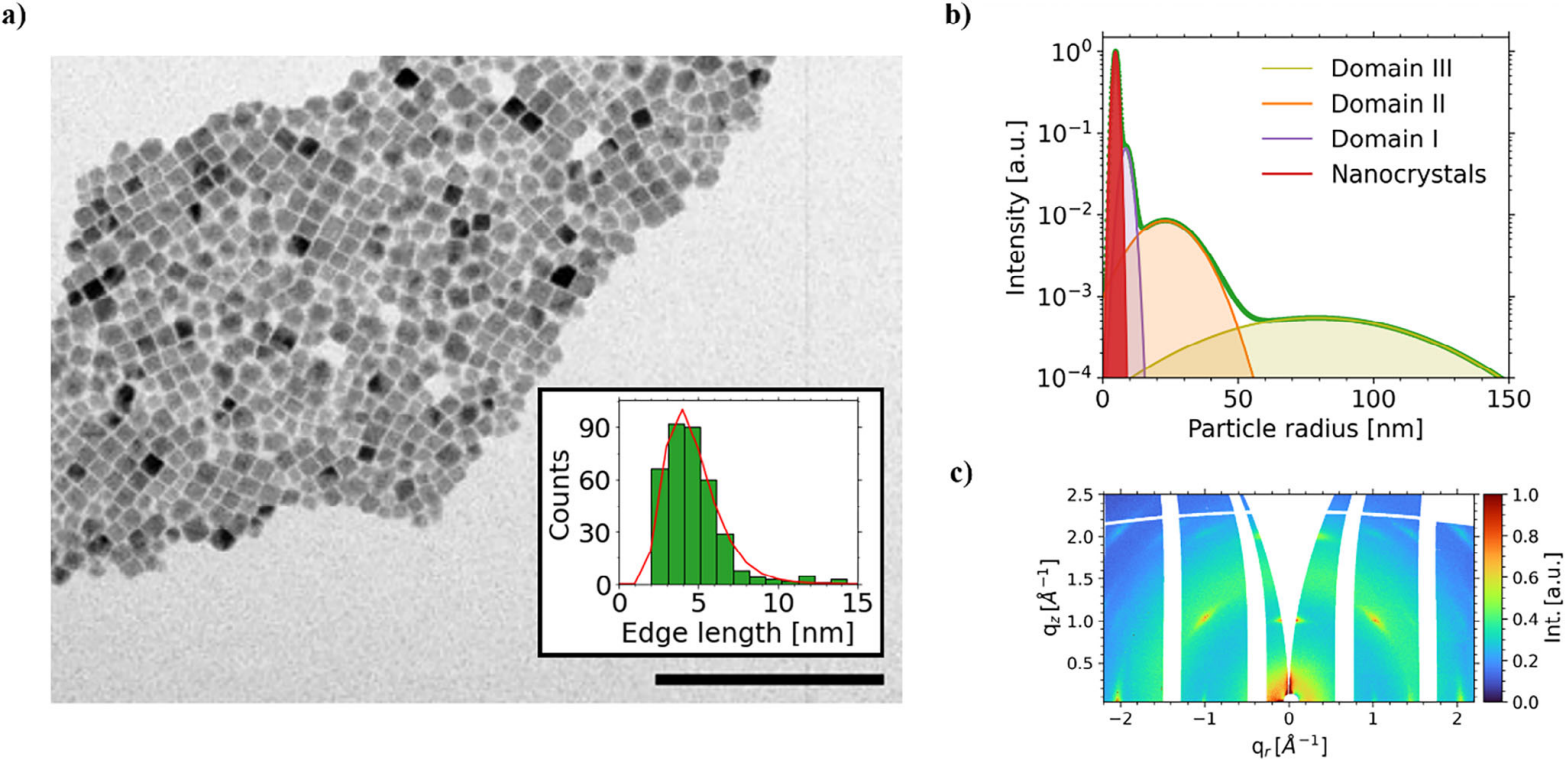
a) TEM image of the CsPbBr_3_ NCs with a scale bar of 200 nm. b) Particle radius distribution obtained from static GISAXS measurements on the seed crystal layer. c) 2D GIWAXS data of the seed crystal film.

Colloidal CsPbBr_3_ NCs were synthesized using the typical hot‐injection method, as described in the Supporting Information.^[^ ^36^ ^]^ Transmission electron microscopy (TEM) images (**Figure** **1**a) reveal their cubic morphology with an average edge length of 4.6 ± 1.7 nm. Upon purification of the as‐synthesized NCs, as described in the supporting information, a colloidal ink of CsPbBr_3_ NC seeds (Figure S1a, Supporting Information) with a concentration of 10 mg mL^−1^ was printed with a slot‐die coater onto 75 × 25 mm glass substrates (Figure S1b, Supporting Information). The printed thin films undergo an additional plasma treatment to remove as many surface ligands from the NCs as possible.^[^ ^37^ ^]^ The horizontal line cut from the grazing incidence small‐angle X‐ray scattering (GISAXS) measurements (Figure S2, Supporting Information) reveals that most scatterers of the seed crystal templated substrate are individual particles with an average radius of 4.7 ± 1.0 nm (structure 1) (Figure 1b). The findings from the GISAXS data are in good agreement with the average NC edge length of 4.6 ± 1.7 nm obtained from the TEM images. Their center‐to‐center distance corresponds to 10.2 ± 1.0 nm, indicating a closely packed structure of individual nanocubes. Besides the smallest scattering structure, three larger domains with average particle radii of 8.5 ± 2.0 nm (structure 2), 23 ± 11 nm (structure 3), and 79 ± 37 nm (structure 4) are modeled to the horizontal line cut. Their respective center‐to‐center distances correspond to 29.0 ± 8.4 nm, 127.0 ± 48.3 nm, and 453 ± 177 nm. We attribute these structures to clusters formed during the coating and plasma treatment procedures.^[^ ^38^ ^]^ Indeed, AFM measurements on the seed crystal layers reveal the existence of closely packed NCs, which we attribute to such cluster formations (Figure S3a,b, Supporting Information) that increase in size and amount with increased NC colloidal ink concentration (Figure S3c,d, Supporting Information). Furthermore, GIWAXS measurements of the seed crystal templated substrate reveal a crystalline film characterized by discrete, lens‐shaped diffraction spots, indicating a highly oriented structure with the (001) plane parallel to the substrate (“face‐on”) as reflected in the GIWAXS azimuthal location of the {100} Bragg peaks ≈q_r_ = 1.0 nm^−1^ (Figure 1c). Due to the amorphous characteristic of the underlying substrate, a broad Debye‐Scherrer ring between 1.3 and 2.3 nm^−1^ is also observable.

### 2.2 CsPbBr_3_ Nanocrystal Seed‐Induced Crystallization Kinetics

#### 2.2.1 Structure Evolution During Printing

In situ GIWAXS measurements are carried out to resolve differences in the crystallization kinetics between seeded and unseeded (control) films. To investigate bottom‐up crystallization in seeded films, a grazing incidence angle of 0.15° is selected. Its calculated X‐ray scattering depth of ≈5 nm allows tracking the formation of the photoactive phase as the crystallization front progresses from the substrate interface toward the film surface. Additionally, the high diffraction intensity at incidence angles near the critical angle of FAPI (0.18°) enables probing lattice interactions with the underlying seed crystals. This configuration also enhances surface sensitivity, allowing detection of weak or early‐stage signals—such as those from seed crystals—that might otherwise be drowned out by the dominant FAPI diffraction. For the control films, an incidence angle of 0.4° is used to probe top‐down crystallization, as expected for FAPI,^[^ ^39^, ^40^ ^]^ given the ≈170 nm scattering depth at this angle. Details of the critical angle calculations are provided in the Supporting Information.

**Figure 2:**
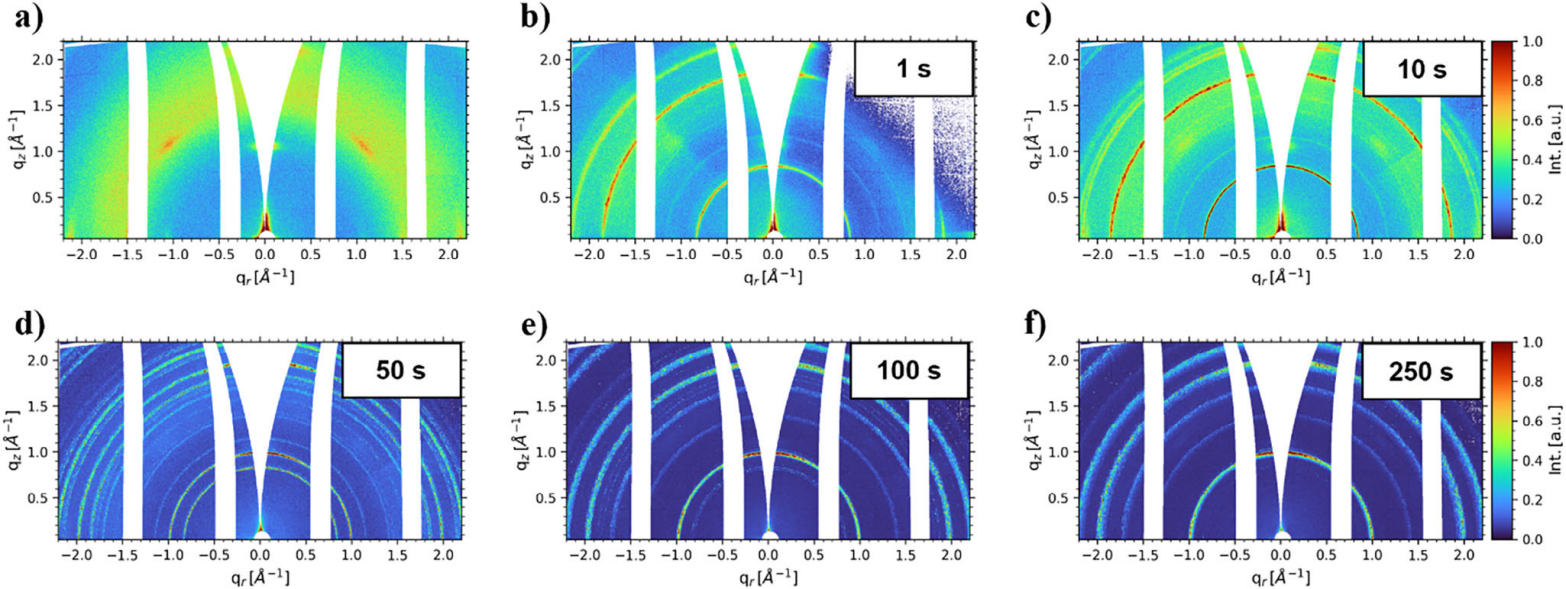
2D GIWAXS data during the perovskite film coating process with seed crystals at selected times: a) prior to deposition, b) 1 s, c) 10 s, d) 50s, e) 100 s, and f) 250 s.

FAPI naturally crystallizes into the photoinactive δ‐phase prior to transitioning to the black photoactive α‐phase via a first‐order solid‐solid phase transition.^[^ ^41^, ^42^, ^43^ ^]^ Such behavior is observed for both seeded and control films, with the δ‐phase crystallizing almost immediately after ink deposition on the substrate. Crystallization occurs almost immediately after deposition, with no significantly prolonged drying stage observed. We attribute such fast crystallization of the photoinactive phase to the quick solvent drying kinetics induced by the utilized N_2_ air‐blade that proves to be highly compatible with solvents possessing a large vapor pressure.^[^ ^11^, ^12^, ^44^ ^]^ 2D GIWAXS data during the coating process of the seeded film are shown in **Figure** **2**. Although the dominant crystalline phase is the δ‐phase at the initial stages of film formation, weak diffraction signals from the CsPbBr_3_ NC layer (Figure 2a) are still observable during the coating process (Figure 2b,c), showcasing their stability after FAPI printing. During the phase transition to the photoactive black phase, the relative intensity of the δ‐phase signals diminishes, while the α‐phase diffraction signals exhibit an increase in intensity (Figure 2d–f). At this stage, an increase in crystallite orientation is observable in the seeded thin films as the α‐phase {100} diffraction ring ≈1.0 nm^−1^ exhibits an increase in intensity around an azimuthal angle of 0°. Unlike the seeded thin film, the control film possesses fully isotropic diffraction rings (Figure S4, Supporting Information). Additionally, the diffraction patterns attributed to the CsPbBr_3_ NC layer vanish from the detected signal as the seed crystals evolve into the bulk perovskite. Whole α‐phase diffraction rings throughout the entire azimuthal range are also observable, revealing an isotropically oriented fraction of probed crystallites.

**Figure 3:**
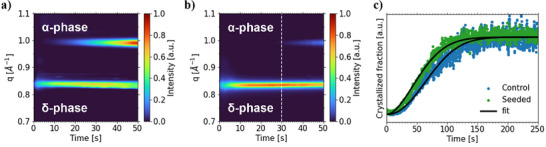
First 50 s of the phase formation kinetics of a) seeded and b) control film as seen in a mapping of the pseudo‐XRD data extracted from the 2D GIWAXS data. c) Normalized JMAK fit (solid line) of the area intensity of the {100} Bragg peaks of seeded (green) and control (blue) film.

Regarding the δ‐to‐α‐phase transition, the seeded thin film transitions earlier to the α‐phase (**Figure** **3**a) compared to the film without CsPbBr_3_ seed crystals, whose phase transition starts at ≈30 s during the coating process (Figure 3b). In situ contour maps spanning a larger *q*‐ & time range for both samples are depicted in Figure S5 (Supporting Information). We rationalize the earlier crystallization onset time in seeded thin films to the bypassing of the nucleation step since the selected NCs act as nuclei that are larger than the critical particle radius for crystal growth.^[^ ^45^ ^]^

To quantify the photoactive phase transition kinetics via the Johnson‐Mehl‐Avrami‐Kolmogorov (JMAK) model,^[^ ^46^ ^]^ the area intensity of the {100} Bragg peaks over time are extracted. The resulting fits (Figure 3c) present an increase of the crystallization rate constant *k* for the seeded film, which is more than six times higher than that of the control film, displaying an accelerated crystal growth rate. The exact values extracted from the JMAK fit can be found in Table S1 (Supporting Information).

##### Competitive Growth Process

**Figure 4:**
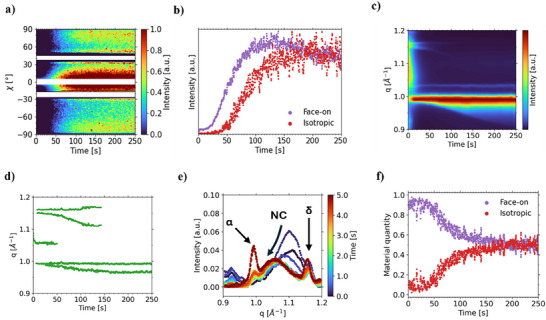
a) Mapping of the azimuthal cuts at the {100} diffraction peak of the 2D GIWAXS data for seeded film printing, highlighting the preferential (001) crystallographic orientation. b) Intensity‐time plots of the oriented “face‐on” and isotropically oriented fraction in the seeded thin film. c) Normalized phase formation kinetics of the seeded film as seen in a mapping of the pseudo‐XRD data extracted from the 2D GIWAXS data. d) Peak position evolution of the respective phases involved in the deposition process. e) In situ GIWAXS pseudo‐XRD diffractograms of the first 5.0 s into the deposition process at selected q‐range. f) Relative material quantity of “face‐on” oriented and isotropically oriented crystallites in the seeded thin film.

Azimuthal tube cuts (tube cuts) around the {100} peak position reveal the respective crystallographic texture.^[^ ^47^, ^48^ ^]^ The control film possesses fully isotropically oriented crystallites, which do not change in regards to their orientation distribution during the probed time (Figure S6, Supporting Information). In the seeded film on the other hand, a strong intensity contribution of the {100} diffraction ring is reflected in a pronounced peak around an azimuthal angle of 0°, surpassing the intensities associated with the isotropic crystallites. Hence, the seeded film is characterized by a preferential orientation with the (001) plane oriented parallel to the substrate (face‐on) (**Figure** **4**a), which is dictated by the orientation distribution of the CsPbBr_3_ seed NCs. Indeed, the highly oriented face‐on signal from the seed crystal layer is also observable from the beginning of the measurement (Figure 4b), as indicated by the non‐zero intensity at the initial stage of the “face‐on” oriented diffraction intensities. Conclusively, the bulk perovskite adopts the templated orientation induced by the seed crystals, suggesting crystal growth following an epitaxial “bottom‐to‐top” growth mechanism from the seeds toward the thin film surface.

The face‐on crystallites also grow much quicker compared to their isotropic counterparts with a crystallization onset time of ≈20 s. Given the discrepancy in the growth onset times between face‐on and isotropic crystals in the seeded thin film, it can be concluded that crystallite formation arises from a competitive growth process, with CsPbBr_3_ NC seeds and randomly formed nuclei both contributing to the overall growth. It is worth noting that the isotropic crystals within the seeded thin film begin to form later than the oriented ones, with their growth onset occurring ≈50 s into the coating process, which is ≈20 s later than in the control film. The delayed growth onset time indicates a reduced ion flux to randomly formed nuclei reminiscent of Ostwald ripening.^[^ ^45^ ^]^ Nevertheless, the total crystallization rate constant in the seeded thin film is higher relative to the control film. Therefore, we conclude that the higher *k* is induced by the CsPbBr_3_ NC seeds enabling a fast crystallization during the solid‐solid growth process via bypassing the nucleation step for crystal growth.

To investigate the competitive growth and growth interaction between the α‐phase and the NC seeds, pseudo‐XRD profiles are extracted from azimuthal angles ranging from −10.0° to 10.0°, optimizing the diffraction intensity ratio between the NCs and surrounding phases (Figure 4c). Peak positions corresponding to the competing crystalline phases are identified and plotted (Figure 4d). Shortly after deposition, the NC's lattice parameter increases, reflected in the primary Bragg peak's shift by ≈0.05 Å^−1^ toward lower scattering vector values, indicative of progressive iodide incorporation from the ink into the lattice (Figure 4e). Concurrently, the α‐phase begins to emerge and continuously grows out of the NC seeds, while the δ‐phase also forms, marked by a Bragg reflection at ≈1.15 Å^−1^ (Figure 4e). ≈20 s into the coating process, the δ‐phase reaches maximum intensity before diminishing as it undergoes a solid‐solid phase transition into the α‐phase, proceeding directly on the seed crystals. This transition timing aligns with the onset of seed‐induced face‐on crystallization at ≈20 s. We observe that the α‐phase grows directly from the NC seeds, bypassing the δ‐to‐α‐phase transition and subsequently incorporates material from the surrounding δ‐phase during its growth. At ≈50 s, the α‐phase peak begins to exhibit splitting on the low‐q side, reaching its maximum separation ≈150 s into the coating process (Figure 4d). We attribute this additional α‐phase component to the isotropically oriented crystallites formed through the aforementioned random nucleation events, as its crystallization onset coincides with their initiation. The two photoactive α‐phases exhibit lattice parameters of 6.33 ± 0.02 Å and 6.48 ± 0.02 Å, respectively, reflecting a compositional difference. Specifically, the α‐phase originating from the seed‐mediated growth is richer in its bromide content. Our results demonstrate that the NC seeds not only bypass the nucleation barrier for FAPI growth but also enable direct α‐phase growth without the intermediate formation of the δ‐phase.

To quantify the relative proportions of oriented‐ and isotropic crystallites, each tube cut is Lorentz‐transformed^[^ ^47^, ^48^ ^]^ and the relative contributions from isotropic‐ and oriented crystallites over the film formation process are analyzed (Figure 4f). The seeded thin film initially possesses a diffraction contribution solely from the CsPbBr_3_ NCs. Therefore, the initial total collected diffraction intensity originates from purely face‐on oriented crystals. As the perovskite ink deposition occurs, a fraction of the α‐phase crystallites grow from the CsPbBr_3_ seeds as oriented face‐on crystals while the remaining crystallites grow isotropically resulting in a final film characterized by an equal ratio of face‐on and isotropic crystals. Thus, including a buried bottom seed crystal layer increases the thin film's crystallographic texture. We attribute such induced orientation to the self‐assembly mechanism of the CsPbBr_3_ nanocubes: given their cubic shape, the NCs minimize their individual surface energy when their face, which is the {100} Bragg plane, lies directly on the substrate surface^[^ ^49^, ^50^, ^51^ ^]^ leading to easily fabricated and highly oriented seed crystal layers for templating the orientation of the final bulk perovskite film.

#### 2.2.2 Optical Properties Evolution During Printing

**Figure 5:**
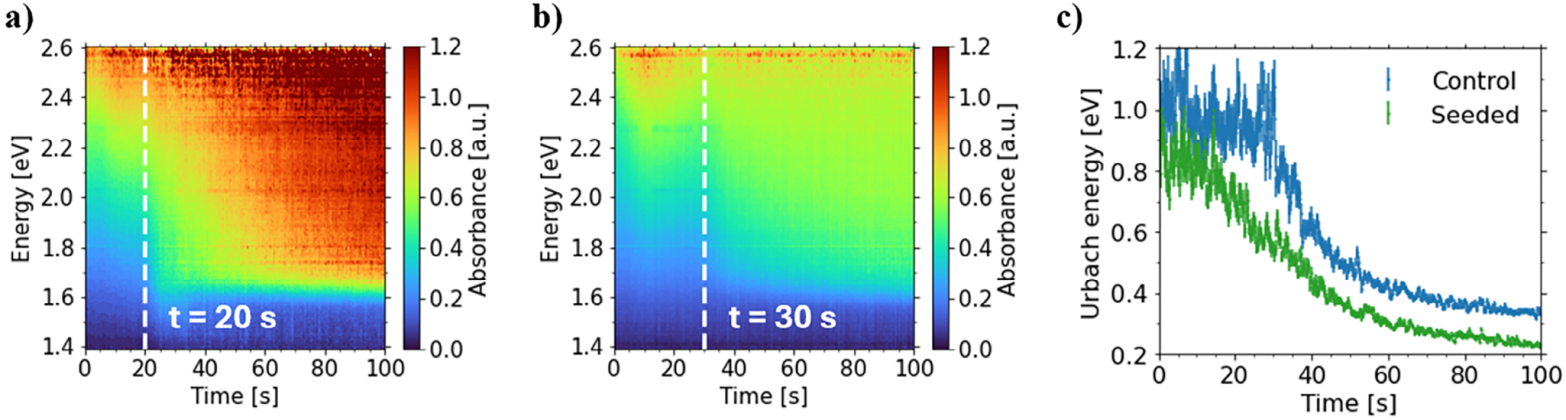
Mapping of in situ UV–vis data of a) seeded and b) control film. c) Urbach energy evolution of seeded (green) and control (blue) film.

The favorable optoelectronic properties of FAPI can be leveraged to track the film formation kinetics from an optical spectroscopic point of view,^[^ ^52^ ^]^ providing information about the film bandgap, absorption properties as well as Urbach energy from the absorption tail.^[^ ^53^ ^]^ In situ transmission mode UV–vis spectroscopy measurements reveal a δ‐to‐α phase transition time of ≈20 s for the seeded thin film (**Figure** **5**a), accompanied by a rapid increase in absorbance for photon energies below 2.20 eV. In comparison, the control film exhibits a phase transition at ≈30 s (Figure 5b). Both phase transition times match well with the in situ GIWAXS data shown in Figure 3a,b. The absorbance increase of the control film for photon energies below 2.20 eV is slower compared to the seeded thin film, with final absorbance values reaching only half of those in the seeded film. The final bandgap of both films after the coating process is estimated via Tauc plot analysis (Figure S7, Supporting Information) to be 1.53 eV for the control film and 1.57 eV for the seeded. The blue‐shifted bandgap for the seeded thin film can be explained by the incorporation of cesium and bromide into the perovskite lattice, effectively forming a mixed halide perovskite.^[^ ^54^, ^55^ ^]^

Furthermore, the thin film thickness is determined to be 820 ± 60 nm for the seeded and 790 ± 110 nm for the control sample via profilometry measurements. With known film thickness values, we extract the Urbach energy **(*E_U_*)** and its temporal evolution over time for both films from their absorption tail shape (Figure 5c). Both films exhibit an initial *E_U_* larger than 0.8 eV followed by an exponential decay in the respective value. The progression of the Urbach energy indicates that as the α phase forms, the band edge becomes more distinct and its defect density decreases. The values for ***E_U_*** were determined to be 230 ± 5 meV and 328 ± 7 meV for seeded and control film, respectively. Further annealing the films at 150 °C for 75 s induces an exponential ***E_U_*** decay to 175 ± 4 meV and 194 ± 5 meV for seeded and control film respectively (Figure S8, Supporting Information), which we attribute to Ostwald ripening‐induced improvement in the crystallinity. The lower ***E_U_*** value in the seeded thin film compared to the control film suggests a reduction in defect density induced by the CsPbBr_3_ NCs.

#### 2.2.3 Phase Evolution During Printing

**Figure 6:**
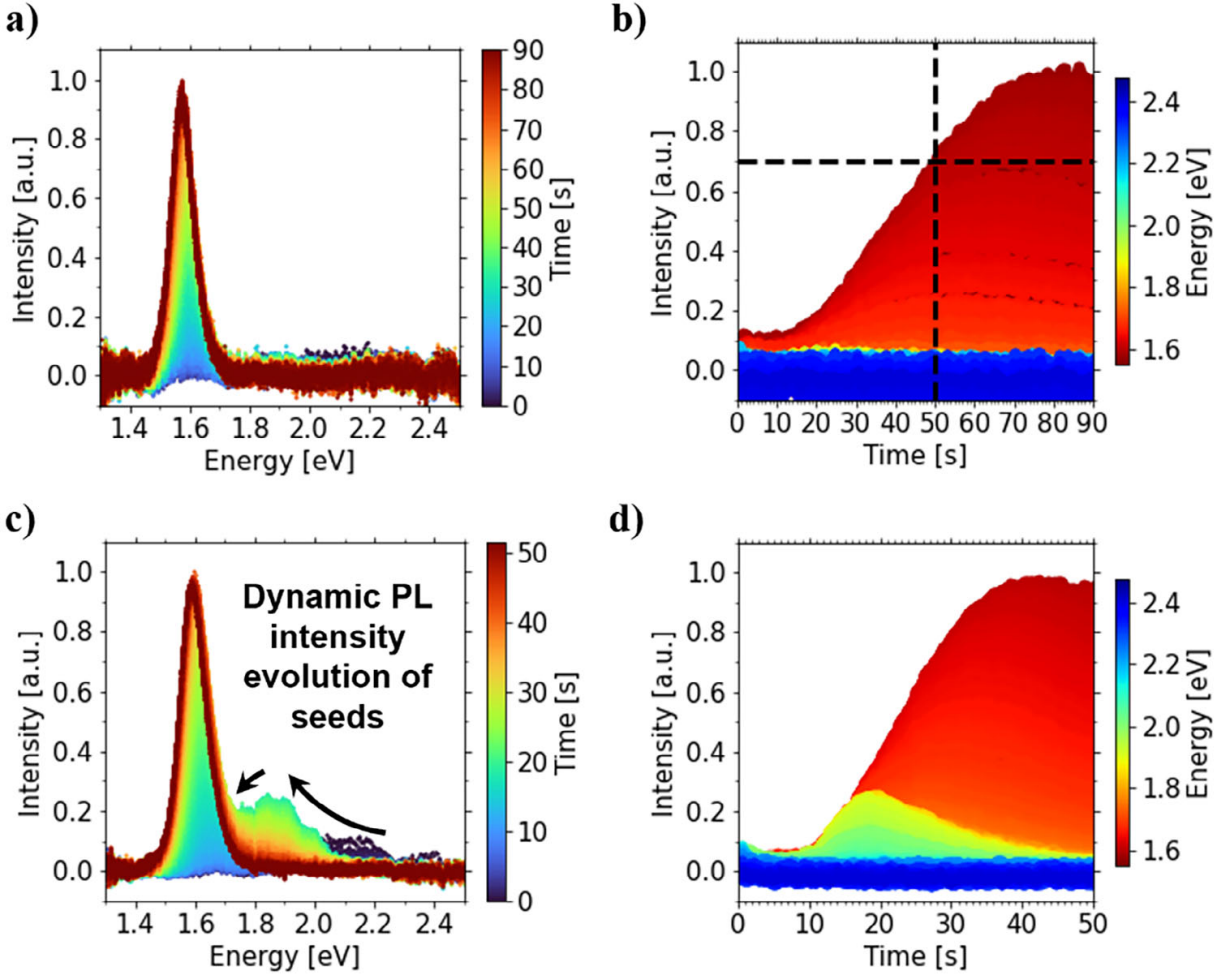
In situ PL maps of printed thin films showing the temporal evolution of PL spectra for (a) control and (c) seeded film, along with the temporal PL emission energy intensity changes for (b) control and (d) seeded film.

Complementary to in situ UV–vis spectroscopy, in situ photoluminescence (PL) spectroscopy was also carried out to obtain more insights about the nuclei during film formation. Our measurements on the control film (**Figure** **6**a) reveal a phase formation involving a PL red‐shift to 1.57 eV associated with the growth of the nucleated phase to the final bulk perovskite.^[^ ^56^ ^]^ Hereby, the PL emission reaches its maximum intensity within 90 s, which is 40 s later than the maximum intensity observed in the seeded film. The respective control film PL intensity at 50 s corresponds to ≈70% of its maximum intensity as marked in dashed lines in Figure 6b, showcasing a slower phase formation rate. The seeded film exhibits two distinct PL signals (Figure 6c): A low‐energy PL emission attributed to an iodide‐rich phase associated with the bulk perovskite and a high‐energy, bromide‐rich PL emission associated with the seeds. Indeed, the bulk perovskite PL emission exhibits a continuous red shift from 1.67 to 1.58 eV. The seed crystal emission follows a similar pattern with a PL red‐shift from ≈1.90 to 1.62 eV. Given the initial PL emission of the CsPbBr_3_ NCs at 2.40 eV (Figure S9, Supporting Information), the seed crystal‐associated PL emission rapidly shifted to 1.93 eV during the δ‐phase formation, followed by a more gradual red‐shift toward the bulk PL emission during the δ‐to‐α‐phase transition. We attribute the initial abrupt red‐shift of the seed crystal PL to a fast halide exchange with its δ‐phase matrix toward iodide‐rich compositions.^[^ ^54^, ^55^ ^]^ The slower red‐shift observed during the solid‐solid phase transition is attributed to the combined effects of continued halide exchange and the growth of iodide‐based perovskites on the bromide‐containing seed crystals.^[^ ^54^, ^55^, ^57^ ^]^ As iodide‐rich perovskites continue to grow on bromide‐rich seeds, the overall bromide‐to‐iodide ratio in the photoactive film decreases significantly. This is evidenced by the reduced PL emission intensity of the seed crystals (Figure 6d) and a continuous red‐shift in their emission spectrum. To investigate the competitive growth and growth interaction in the seeded film, we extract the PL emission energy and corresponding linewidth for both the seeded and control films at the respective time points of maximum emission intensity (Figure S10a,b, Supporting Information). The PL emission of the seeded film exhibits a blue‐shift relative to the control, indicating bromide incorporation into the perovskite structure. Additionally, the increased linewidth of the seeded film emission suggests a higher degree of energetic disorder, consistent with disordered halide alloying reminiscent of mixed halide perovskites. Interestingly, the PL emissions from seeded and control measurements exhibit intensities prior to the phase transition onset times of ≈20 and ≈30 s for seeded and control films that are determined from in situ GIWAXS and UV–vis measurements. In fact, the seed crystal PL emission maximum intensity, as highlighted by the green graph in Figure 6d, is reached at the previously determined phase transition onset time. Therefore, we attribute the PL emissions prior to each phase transition onset to the emission of nuclei that possess a higher PL quantum yield than bulk perovskite crystals.^[^ ^58^, ^59^, ^60^ ^]^

A crucial aspect for successful NC‐induced seeding is the removal of surface ligands attached to the respective NCs, as they limit further crystal growth, diminishing their role as nucleation seeds for bulk perovskite films.^[^ ^61^, ^62^, ^63^ ^]^ For this purpose the NC seed layer underwent a short plasma treatment procedure prior to FAPI coating to remove as many surface ligands as possible.^[^ ^37^ ^]^ In situ PL spectroscopy measurements on FAPI growing on a NC layer without plasma treatment demonstrate a similar growth behavior to plasma‐treated, seed‐induced FAPI; albeit with a significantly lower bulk perovskite PL emission intensity (Figure S11a, Supporting Information). Whilst a bulk perovskite is growing, the stagnation in PL emission suggests a surface ligand‐induced increase in the structure's defect density, likely caused by more disordered growth and diminished crystallinity. In contrast, the PL intensity of NCs with surface ligands remains comparable to that of plasma‐treated NC films, as evidenced by the similar intensity evolution near 1.90 eV (Figure S11b, Supporting Information), demonstrating that the NCs maintain their optical properties after the plasma treatment procedure. Comparing the 2D GIWAXS data of a plasma‐treated seed crystal film (Figure 1c) to an untreated CsPbBr_3_ NC film (Figure S12, Supporting Information) shows a reduced degree of preferred orientation in the plasma‐treated film, reflected in the azimuthal spreading of the diffraction signals. Nevertheless, the plasma‐treated films still exhibit a high degree of “face‐on” orientation and phase stability as no additional diffraction peaks are observed.

### 2.3 Film Formation During Printing

**Figure 7:**
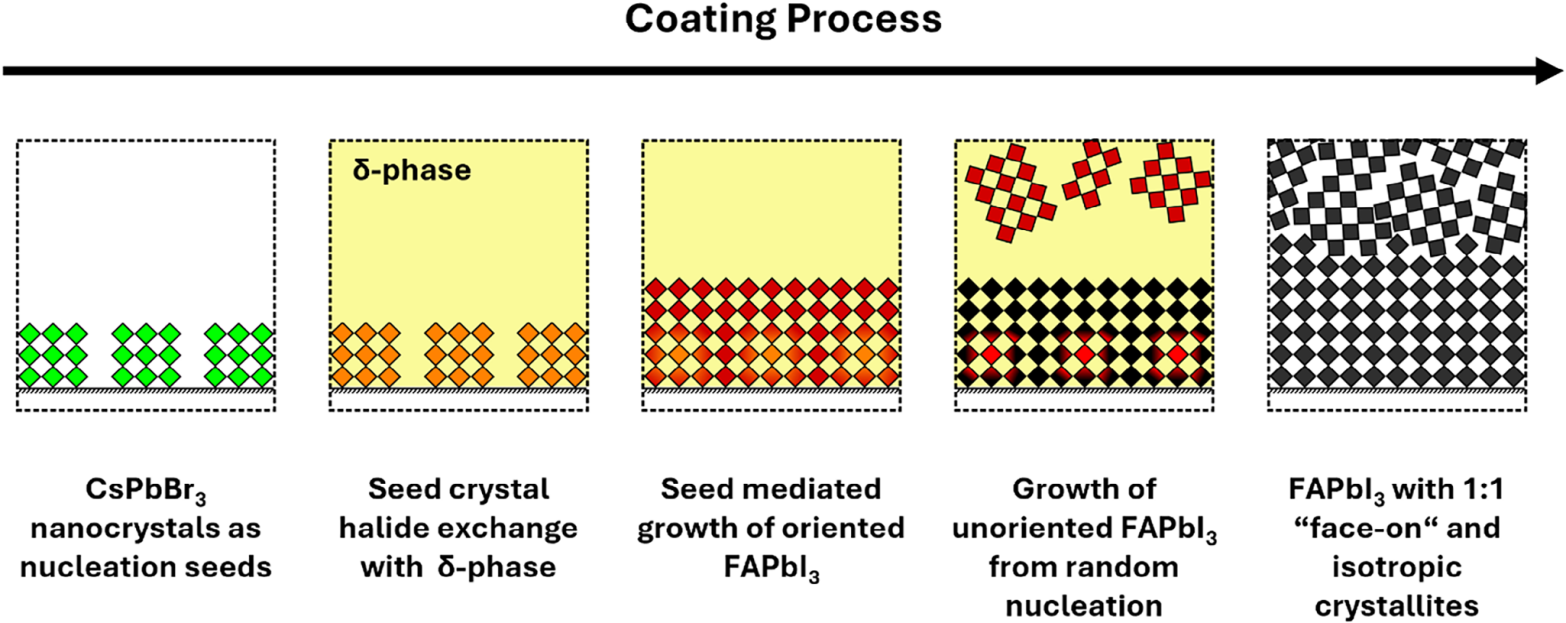
Schematic depiction of the buried bottom seed mediated thin film formation as obtained from the multimodal in situ analysis during printing of the FAPI film.

Combining the results obtained from in situ‐GIWAXS, ‐UV–vis, and – PL measurements during printing, we highlight the major processes occurring during the film formation of α‐FAPI (**Figure** **7**): The initial seed crystal layer consisting of closely packed CsPbBr_3_ NCs remains structurally intact as shown in Figure 2. Upon ink deposition, the photoinactive δ‐phase rapidly crystallizes due to air‐blade induced accelerated evaporation of solvents and undergoes a fast halide exchange with the seed crystals as reflected in the red‐shift of the seed crystal PL emission shown in Figure 6. The induced halide exchange has a beneficial effect on the thin film growth process as the lattice mismatch between CsPbBr_3_ NCs and α‐FAPI decreases. Throughout the deposition process, both seed crystal and grown bulk perovskite are continuously red‐shifting their PL emission, indicating further halide exchange during crystal growth. In situ GIWAXS and UV–vis results describe a phase transition onset time of ≈20 s for the δ‐to‐α transition of “face‐on” oriented crystallites dictated by the orientation of the seed crystals. However, selected azimuthal cuts reveal that the α‐phase initiates growth directly from the NC seeds, which act as anchoring sites for the δ‐to‐α phase transition. Randomly oriented crystals begin to form ≈30 s after the growth onset of “face‐on” oriented crystallites due to the competitive growth process between seeded crystals and crystals from randomly formed nuclei resulting in an overall equal ratio of “face‐on”‐ and isotropically oriented crystals. JMAK equation fits of seeded and control samples show that the seeded thin film exhibits crystallization kinetics equivalent to those of an unseeded film with an ≈10% lower activation energy for crystallization. Additionally, compared to films without seed crystals, the seeding approach results in a marked increase in thin‐film absorbance and a reduced defect density, as evidenced by in situ UV–vis measurements and Urbach energy analysis. The final coated film exhibits a bandgap of 1.57 eV, attributed to bromide and cesium incorporation into the crystal structure.

## 3 Conclusion

In conclusion, we present an in‐depth, multimodal in situ study on the beneficial effects of incorporating a bottom seed layer consisting of CsPbBr_3_ NCs on the film formation of printed FAPI using slot‐die coating. Utilizing such seeds enables the bypassing of the nucleation step during the δ‐to‐α phase transition, leading to faster crystal growth of the photoactive α phase. Besides such faster crystal growth, the seed crystals' natural “face‐on” orientation due to their cubic shape results in the “face‐on” orientation of the printed perovskite film. The combined effects of a faster crystal growth rate and templated orientation lead to more control during the processing of lead halide perovskites, resulting in the production of scalable thin films with reduced defect densities. This study enhances the understanding of perovskite thin film growth mechanisms, particularly in the context of buried bottom seeding, and provides valuable insights for optimizing this approach in future device applications.

## Conflict of Interest

The authors declare no conflict of interest.

## Author Contributions

A.B.‐A. wrote the original draft, conceptualized the study, conducted formal analysis, investigation, and validation. J.F. performed measurements and investigation (in situ UV–vis, PL). X.C. carried out AFM measurements and contributed to GIWAXS and GISAXS measurements. T.B. and L.L. contributed to the GIWAXS and GISAXS measurements. M.S. and S.K.V. provided resources (beamtime for GIWAXS and GISAXS). D.G. conducted TEM measurements, and L.P. provided resources for TEM. P.M.‐B. conceptualized the study, reviewed and edited the manuscript, supervised the work, provided resources, administered the project, and acquired funding.

## Supporting information



Supporting Information

## Data Availability

All relevant data are included in the paper and its Supplementary Information. The data can also be found at the following public repository: https://mediatum.ub.tum.de/XX.
