# Supplementary material for: CsPbBr3 Nanocrystals as Bottom Interface Nucleation Seeds for Printing Oriented FAPbI3 Thin Films: An In Situ Study
Source: Small. 2025 Aug 20;21(39):e05895. doi: 10.1002/smll.202505895 (PMC12490184; doi:10.1002/smll.202505895)
Supplement: Supplementary file 1 — Supporting Information [file SMLL-21-e05895-s001.pdf]

NANO · MICRO
small

Supporting Information

for *Small*, DOI 10.1002/smll.202505895

$CsPbBr_3$ Nanocrystals as Bottom Interface Nucleation Seeds for Printing Oriented $FAPbI_3$ Thin Films: An In Situ Study

*Altantulga Buyan-Arivjikh, Jascha Fricker, Thomas Baier, Xiaojing Ci, Lixing Li, Deepika Gaur, Lakshminarayana Polavarapu, Matthias Schwartzkopf, Sarathlal Koyilot Vayalil and Peter Müller-Buschbaum**

Supporting Information

CsPbBr$_3$ Nanocrystals as Bottom Interface Nucleation Seeds for Printing Oriented FAPbI$_3$ Thin Films: An In-Situ Study

Altantulga Buyan-Arivjikh[1], Jascha Fricker[1], Thomas Baier[1], Xiaojing Ci[1], Lixing Li[1], Deepika Gaur[2], Lakshminarayana Polavarapu[2], Matthias Schwartzkopf[3], Sarathlal Koyiloth Vayalil[3,4], Peter Müller-Buschbaum[1,*]

[1]Chair for Functional Materials, Department of Physics, TUM School of Natural Sciences, Technical University of Munich, James-Franck-Straße 1, 85748 Garching, Germany

[2]Department of Physical Chemistry, CINBIO, Universidade de Vigo, Materials Chemistry and Physics Group Campus Universitario As Lagoas, Marcosende, 36310 Vigo, Spain

[3]Deutsches Elektronen-Synchrotron DESY, Notkestraße 85, Hamburg 22607, Germany

[4]Department of Physics, Applied Science Cluster, UPES, Dehradun 248007, India

*Corresponding author: muellerb@ph.tum.de

Materials

All chemicals were used as purchased without further purification or optimization procedures. Formamidinium iodide (FAI, ≥98 % purity), lead(II) iodide (PbI_2, 99.999 % purity), lead(II) bromide ($PbBr_2$, 99.999 % purity), methylammonium chloride (MACl, 98 % purity), cesium carbonate ($CsCo_3$, 99 % purity), 2-Methoxyethanol (2-ME, 99.8 % purity), 1-Methyl-2-pyrrolidinone (NMP, 99.5 % purity), ethyl acetate (EtOAc, 99.8 % purity), hexane (≥99 % purity), octane (98 % purity), 1-octadecene (technical grade), oleylamine (technical grade) & oleic acid (90 % purity) were purchased from Sigma Aldrich.

$CsPbBr_3$ nanocrystal synthesis

$CsPbBr_3$ nanocrystals (NCs) were prepared via a modified method described by Martinez *et al.* [1]. In short, a $PbBr_2$ solution was prepared by dissolving 166 mg of $PbBr_2$ with 1.5 ml of oleylamine and 1.5 ml of oleic acid in 12 ml of 1-octadecene at 150 °C in a 40 ml glass vial. A second solution containing 16.28 mg $CsCO_3$ with 50 µl of oleic acid was prepared in 0.8 ml of 1-octadecene under 150 °C in a 4 ml glass vial and was kept at 100 °C for further use. 6 ml of the $PbBr_2$ containing solution was heated to 175 °C under continuous stirring (700 rpm) using a magnetic stirrer while heating. The stirring rate was increased to 1000 rpm once the solution reached the desired temperature. 400 µl of the $CsCo_3$ containing solution was swiftly injected into the hot solution, followed by a 5 s waiting time. Afterward, the solution containing a glass vial was put under an ice bath.

Nanocrystal purification and seed crystal ink preparation

The NC solution was mixed with equal volumes of EtOAC and centrifuged at 8000 rpm for 10 minutes. Afterwards, the supernatant was removed from the solution and the sediment was redispersed in 5 ml of hexane, followed by a second centrifugation step at 5000 rpm for 8 minutes. The respective supernatant was transferred to a glass vial and put inside a vacuum chamber to evaporate the solvent. Octane was added to the NC containing vial to achieve a nanocrystal concentration of 10 mg/ml. Finally, the solution was filtered into another glass vial using a 0.2 µm PTFE syringe filter from Millex.

$FAPbI_3$ solution preparation

0.88 M of FAI and 0.8 M of PbI_2 were dissolved in 5 ml 2-ME at room temperature until dissolved. The solution was then heated to 120 °C under continuous stirring for 30 minutes to form $FAPbI_3$ powder[2-4]. The powder was then filtered from the solution followed by a drying

& annealing step for 30 minutes at 150 °C in a drying oven. The $FAPbI_3$ powder was further utilized to form a 1 molar solution of $FAPbI_3$ in a mixture of 2-ME (95 vol %) and NMP (5 vol %) with 35 mol % of MACl. Finally, the solution was filtered to another glass vial using a 0.2 μm PTFE syringe filter from Millex.

Thin film fabrication

Glass substrates (75 mm × 25 mm) were used for slot-die coating. Prior to deposition, the substrates were sequentially cleaned in an ultrasonic bath for 15 minutes each in a 2% aqueous Hellmanex III solution, deionized water, acetone, ethanol, and isopropanol. After the final rinse, substrates were dried with nitrogen and treated in an ozone plasma chamber for 10 minutes at 250 W to enhance surface wettability and remove residual contaminants.

Both the $CsPbBr_3$ seed crystal layer and the $FAPbI_3$ layer were deposited under atmospheric conditions using slot-die coating at a print speed of 10 mm/s, a pump rate of 10 μL/s, and a printing height of 200 μm. For the seed layer, substrates were preheated to 30 °C before deposition of the $CsPbBr_3$ nanocrystal solution. The resulting film underwent an additional plasma treatment (250 W, 18 s) to remove excess surface ligands and further improve wettability [5].

For the $FAPbI_3$ layer, either blank glass (control) or seed-templated substrates were preheated to 40 °C before coating. A nitrogen air blade (25 L/min) was employed during slot-die coating to accelerate solvent evaporation [6, 7] and improve film uniformity through air-blade-assisted smoothing.[6, 8]

All coating and annealing steps were conducted under ambient atmosphere. Films were annealed at 150 °C during in-situ UV-Vis measurements to track crystallization under representative thermal conditions. Additional details on the slot-die coating system are provided in [9].

Characterizations

Grazing Incidence Wide/Small Angle X-ray scattering (GIWAXS/GISAXS)

GIWAXS/GISAXS measurements were carried out at the P03 MiNaXS[10] beamline at the PETRA III synchrotron DESY, Hamburg using a photon energy of 11.87 keV. GIWAXS signals were collected with a LAMBDA 9M detector (X-Spectrum) with a sample-to-detector-distance (SDD) of 209 mm and exposure times of 1.0 s, 0.2 s, and 0.1 s as well as incidence angles of 0.15 °, 0.15 °, and 0.4 ° for static, *in-situ* seeded, and *in-situ* control samples respectively.

To minimize beam-induced damage, each frame was recorded at a fresh location on the substrate with a maximum counting time well below the threshold for radiation damage being individually determined before. The X-ray beam was systematically translated opposite to the direction of the slot-die head movement, such that each measurement position corresponded to a defined time point after ink deposition. This configuration enabled a spatial-to-temporal mapping of the crystallization dynamics during film formation.
GISAXS signals were collected with a Pilatus 2M detector (Dectris) with an SDD of 4160 mm, an exposure time of 1.0 s, and an incidence angle of 0.4 °. Image conversion and data extraction were carried out using the Python tool INSIGHT [11].

In-situ UV-Vis spectroscopy

In-situ UV-Vis spectroscopy measurements were carried out using a CAS140 CT-154 compact array spectrometer (Instrument Systems) in transmission mode with an integration time of 200 ms. A broadband LED (GoldLED375, Dr. Licht GmbH) was installed underneath the substrate to act as a light source.

In-situ PL spectroscopy

In-situ PL spectroscopy measurements were carried out using a CAS140 CT-154 compact array spectrometer (Instrument Systems) with an integration time of 200 ms and a continuous wave excitation laser of 450 nm (CSP450, Thorlabs). A long-pass filter was installed in front of the detection optics to filter out the light signal originating from the excitation source.

Film thickness measurement

Film thicknesses were measured using a stylus profilometer (DektakXT, Bruker). Two parallel scratches (~1 mm apart) were introduced through the full film thickness down to the substrate. The resulting step profiles were recorded, and the average height difference across the measured region was taken as the film thickness.

Transmission Electron Microscopy (TEM)

TEM images were obtained with a JEOL JEM 1010 transmission electron microscope operating at an acceleration voltage of 100 kV.

Atomic Force Microscopy (AFM)

Atomic Force Microscopy (AFM) measurements were carried out on a FlexAFM (Nanosurf). The corresponding images were processed using the software package Gwyddion[12].

Data analysis

GIWAXS orientation analysis

Azimuthal tubecuts were extracted between 0.9 Å^{-1} and 1.1 Å^{-1}. Horizontal lines were fitted to the data for isotropic contributions to the signal whereas face-on contributions were fitted using Gaussian curves with respective center positions at azimuthal angles of -90 °, 0 ° and 90 °. The extracted parameters were applied for the Lorentz correction procedure [13, 14].

Johnson-Mehl-Avrami-Kolmogorow (JMAK) fits

JMAK curve fits were carried out on the areal intensity of the in-situ GIWAXS [13] Bragg peaks. The following equation was utilized for the fit[15]:

$$X(t) = 1 - exp(kt^n)$$

where *X(t)* is the relative amount of the crystallized phase at time *t*, *k* is the crystallization rate constant, and *n* is the Avrami exponent.

The crystallization rate constant is described via the Arrhenius equation:

$$k \propto exp\left(-\frac{E_a}{RT}\right)$$

with *R, T* & E_a corresponding to the universal gas constant, temperature, and activation energy for crystallization. Comparing the rate constants of seeded and control films, one can obtain the change in activation energy ΔE_a via:

$$\Delta E_a = RTln\left(\frac{k_{Control}}{k_{Seeded}}\right)$$

Lattice parameter determination

Cubic symmetry for the photoactive phases was assumed and the interplanar distance *d* of the {100} Bragg peaks was equated to the lattice parameter via:

$$d = \frac{2\pi}{q}$$

Critical angle calculation

The critical angle α_c is calculated from the complex refractive index: [13]

$$n = 1 - \delta(\lambda) + i\beta(\lambda)$$

with $\delta(\lambda)$ being the corresponding dispersion factor and $\beta(\lambda)$ the X-ray absorption factor. Both factors were calculated via:[16]

$$\delta(\lambda) = \frac{\rho N_A}{M_a}\frac{r_e \lambda^2}{2\pi}\sum f^{'}$$

$$\beta(\lambda) = \frac{\rho N_A}{M_a}\frac{r_e \lambda^2}{2\pi}\sum f^{''}$$

where ρ is the density of $FAPbI_3$, M_a its molecular mass, r_e the classical electron radius and $f^{'}$ & $f^{''}$ the real- and imaginary part of the atomic scattering factor which were obtained from the table by Henke et al. [17] Structural data for the calculation was obtained from the refined structural data provided by Hanusch et al. [18] The critical angle of $FAPbI_3$ was calculated via:

$$\alpha_c = \sqrt{2\delta(\lambda)}$$

Urbach energy extraction from in-situ UV-vis measurements

From the relation between absorbance and transmission, the following equation holds true:

$$A = -log_{10}T = \alpha d$$

where A is the absorbance, T the sample transmission, α the sample's absorption coefficient, and d the film thickness. Furthermore, the Urbach energy can be obtained from the following relation[19]:

$$ln(\alpha) = \frac{E_{Ph}}{E_U} + const.$$

where E_U is the Urbach energy and E_{Ph} the respective photon energy. It becomes noticeable that the inverse of the Urbach energy is the linear slope of the logarithmic absorption coefficient near the band gap energy. Consequently, the equation was fitted to the slope of the logarithmic absorption coefficient at energies between 1.55 eV and 1.65 eV for all obtained spectra.

In-situ PL spectroscopy

An intensity correction via Jacobian transformation was conducted for all measurements [20]. All *in-situ* PL spectra were normalized to the maximum intensity of the seeded PL film. Peak positions were extracted for the seeded film using a two-Gaussian curve fitting approach: a high-energy peak corresponding to the seed crystal and a low-energy peak associated with the bulk perovskite PL emission. For the control film, a single Gaussian curve was sufficient to

represent the PL emission. Furthermore, areal intensities were calculated from the extracted fitting parameters, whereas the seeded film's intensity is the sum of the bulk- and seed crystal PL emission.

GISAXS modelling

Horizontal line cuts were extracted from the 2D GISAXS data at the Yoneda peak position. The corresponding diffuse scattering factor for monodisperse objects is as follows:

$$P(\vec{q}) \propto N|F(\vec{q})|^2 S(\vec{q})$$

with $F(\vec{q})$, $S(\vec{q})$ being the respective form- and structure factor for N scattering objects. Furthermore, the distorted wave Born approximation (DWBA) was applied to account for multiple scattering events due to the grazing incidence geometry. The DWBA accounts for all possible combinations of scattering and reflection events experienced by the incident and exiting X-ray beams. The respective diffuse scattering vector is then expressed as:

$$P(\vec{q}) \propto N|F_{DWBA}(\vec{q})|^2 S'(\vec{q})$$

We assume the local monodisperse approximation holds for the system. Consequently, the total scattering vector results from the incoherent superposition of differently sized domains [21, 22]. Herein, we model four cylindrical substructures to represent the different orientations of individual nanocubes on the substrate and their corresponding cluster formations, each defined by a radius R and height H, using the following form factor expression:

$$F(\vec{q}) = 2\pi H R^2 \frac{J_1(q_r R)}{q_r R} sin\left[q_z \frac{H}{2}\right] exp\left[i q_z \frac{H}{2}\right]$$

With $J_1(q_r R)$ being the first-order Bessel function of the first kind and qr & qz being the in-plane- and out-of-plane scattering vectors, respectively.

To model the intensity profile of horizontal line cuts and extract centre-to-centre domain spacings within a 1D paracrystalline lattice—accounting for cumulative disorder and resulting loss of long-range order—the Hosemann interference function is employed:

$$S\,(q_y)\; = \frac{1 - exp\,(\pi\sigma_D^2 D^2 q_y^2)^2}{1 + exp\,(\pi\sigma_D^2 D^2 q_y^2)^2 - 2exp\,(\pi\sigma_D^2 D^2 q_y^2)\,cos\,(q_y D)}$$

where σ denotes the standard deviation from the mean center-to-center distance D, assuming a Gaussian distribution. The model comprises a sum of four distinct domains. Model construction and fitting of the recorded scattering data are performed using a custom Python-based script.

Figures and Tables

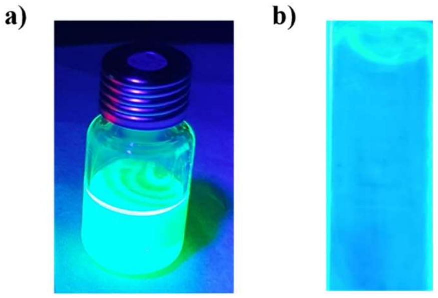

Figure S1 a) Colloidal solution of $CsPbBr_3$ nanocrystals (10 mg ml^{-1}) in octane. b) $CsPbBr_3$ nanocrystal seed layer coated onto a glass substrate.

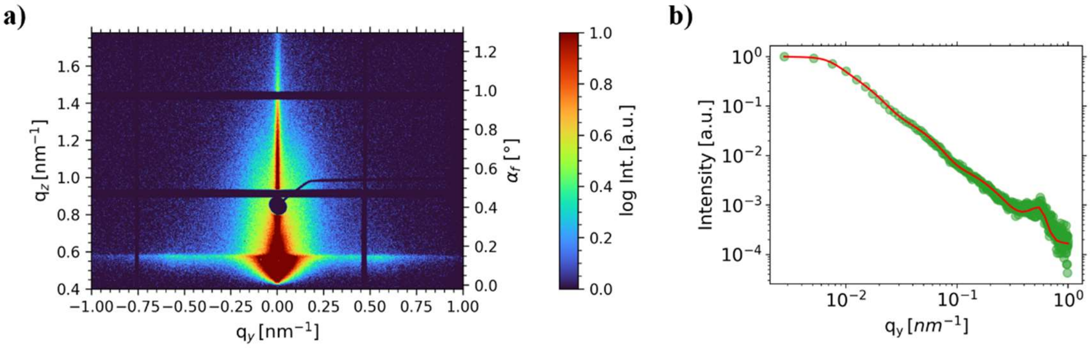

Figure S2 a) 2D GISAXS data of the originally deposited $CsPbBr_3$ nanocrystal seed layer. b) Corresponding horizontal line cut of the 2D GISAXS data taken at the Yoneda region, shown together with a model fit (solid line).

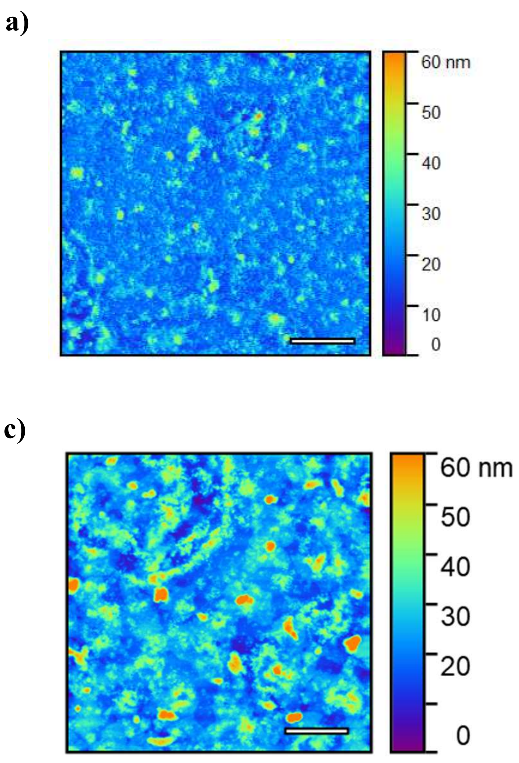

Figure S3 AFM topography images of deposited $CsPbBr_3$ nanocrystal seed layer with a scale bar length and colloidal ink concentration of a) 1 µm, 10 mg/ml b) 200 nm, 10 mg/ml c) 1 µm, 20 mg/ml and d) 1µm, 30 mg/ml.

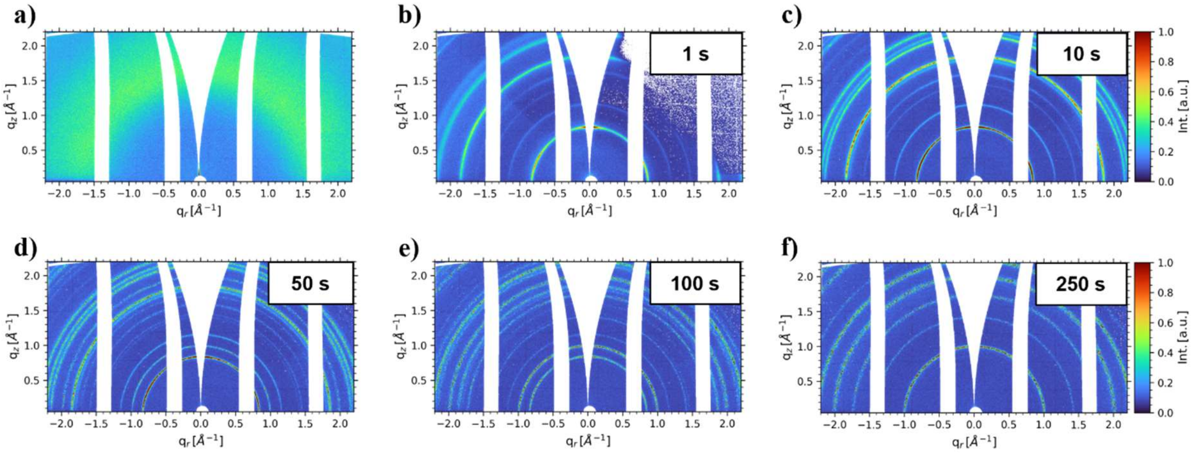

Figure S4 2D GIWAXS data during the perovskite film coating process without seed crystals (control) at selected times: a) prior to deposition, b) 1 s, c) 10 s, d) 50s, e) 100 s & f) 250 s.

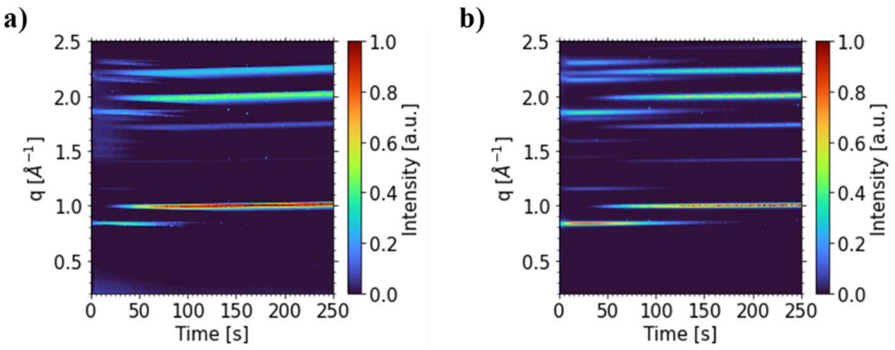

Figure S5 Mapping of pseudo-XRD data extracted from in-situ GIWAXS data measured during printing of a) the seeded and b) the control film.

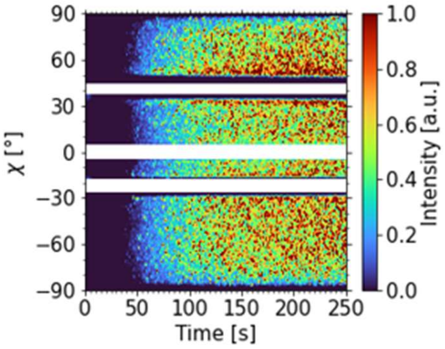

Figure S6 Mapping of azimuthal cuts from the in-situ 2D GIWAXS data of the control {100} diffraction peak highlighting the isotropic crystallographic orientation.

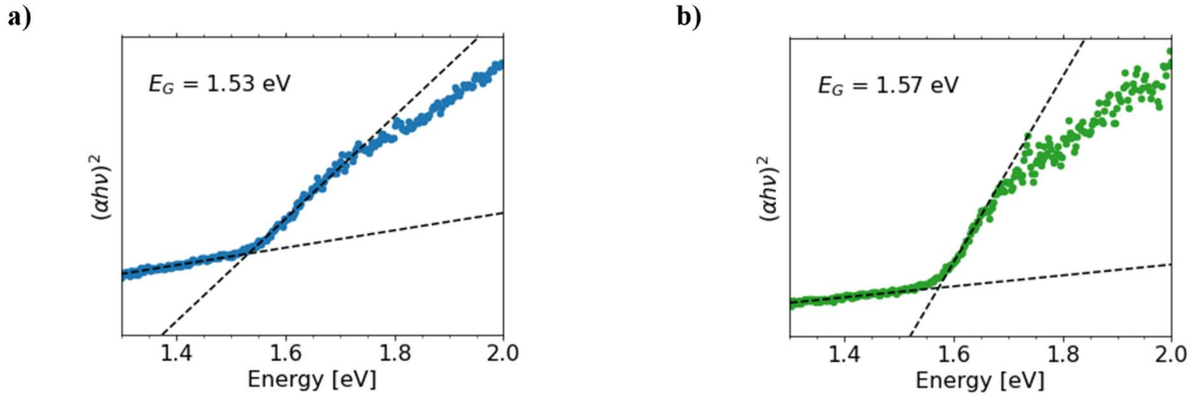

Figure S7 Example of Tauc plots taken at 100 s during the printing process for the a) control and b) seeded sample.

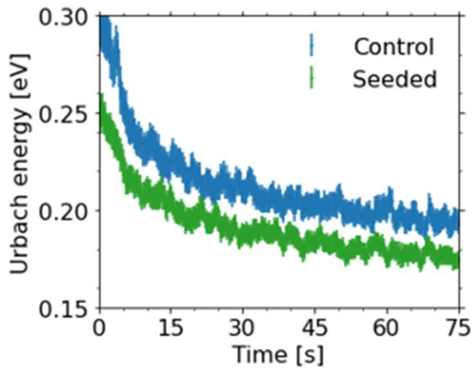

Figure S8 Urbach energy evolution of seeded (green) and control (blue) film during annealing at 150 °C.

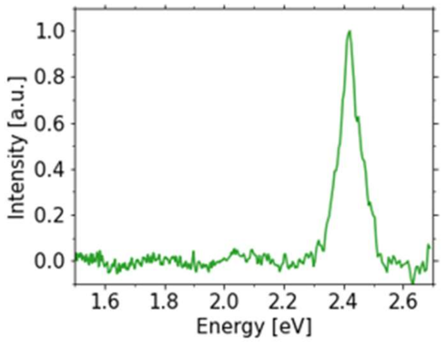

Figure S9 PL spectrum of the $CsPbBr_3$ nanocrystal seed crystal layer.

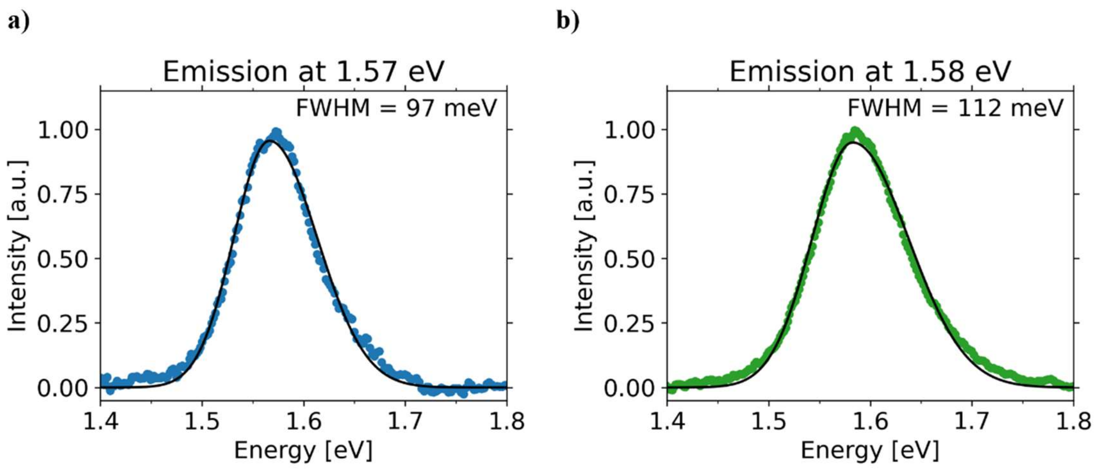

Figure S10 PL spectra and obtained PL emission energy and linewidth of a) control and b) seeded film at their respective timestamp of maximum intensity.

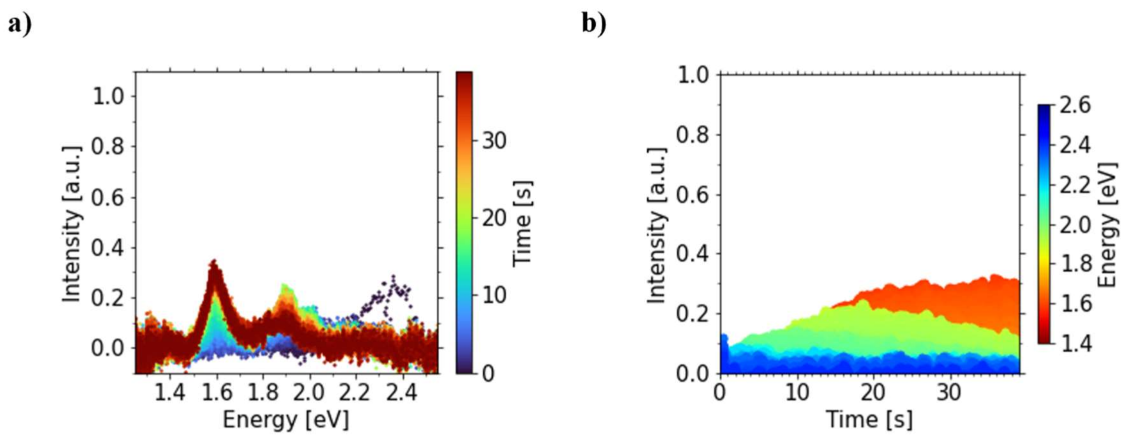

Figure S11 In-situ PL plots of a seeded film without plasma treatment: a) temporal evolution of the PL spectra and b) temporal evolution of PL emission energy intensities.

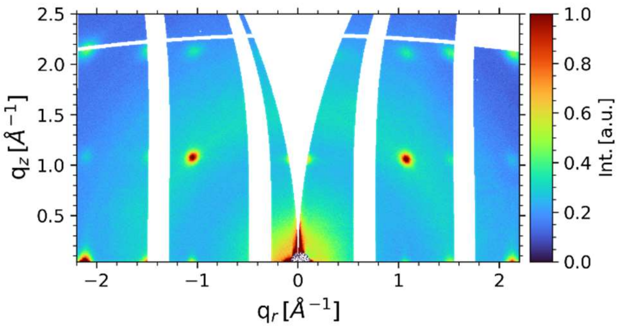

Figure S12 2D GIWAXS data for a $CsPbBr_3$ nanocrystal film without plasma treatment.

Table S1 JMAK fit parameters

Sample	k [s^{-1}]	n
Control	(6.69 ± 0.03) x 10^{-5}	2.17 ± 0.01
Seeded	(43.60 ± 0.30) x 10^{-5}	1.85 ± 0.01

References

[1] C. Otero‐Martínez, D. García‐Lojo, I. Pastoriza‐Santos, J. Pérez‐Juste, L. Polavarapu, *Angewandte Chemie* **2021**, *133*, 26881-26888.

[2] Y. Zhang, Y. Wang, X. Yang, L. Zhao, R. Su, J. Wu, D. Luo, S. Li, P. Chen, M. Yu, *Advanced Materials* **2022**, *34*, 2107420.

[3] M. I. Saidaminov, A. L. Abdelhady, B. Murali, E. Alarousu, V. M. Burlakov, W. Peng, I. Dursun, L. Wang, Y. He, G. Maculan, *Nature communications* **2015**, *6*, 7586.

[4] Y. Xie, Z. Wang, Y. Cui, L. Qiao, K. Chi, D. Shi, Y. Bai, Z. Wu, S. He, *Journal of Solid State Chemistry* **2024**, *334*, 124657.

[5] E. Moyen, A. Kanwat, S. Cho, H. Jun, R. Aad, J. Jang, *Nanoscale* **2018**, *10*, 8591-8599.

[6] D. Burkitt, R. Swartwout, J. McGettrick, P. Greenwood, D. Beynon, R. Brenes, V. Bulović, T. Watson, *RSC advances* **2019**, *9*, 37415-37423.

[7] G. Cotella, J. Baker, D. Worsley, F. De Rossi, C. Pleydell-Pearce, M. Carnie, T. Watson, *Solar Energy Materials and Solar Cells* **2017**, *159*, 362-369.

[8] C. Teixeira, R. Fuentes-Pineda, L. Andrade, A. Mendes, D. Forgács, *Materials Advances* **2023**, *4*, 3863-3873.

[9] M. A. Reus, T. Baier, C. G. Lindenmeir, A. F. Weinzierl, A. Buyan-Arivjikh, S. A. Wegener, D. P. Kosbahn, L. K. Reb, J. Rubeck, M. Schwartzkopf, *Review of Scientific Instruments* **2024**, *95*.

[10] A. Buffet, A. Rothkirch, R. Döhrmann, V. Körstgens, M. M. Abul Kashem, J. Perlich, G. Herzog, M. Schwartzkopf, R. Gehrke, P. Müller-Buschbaum, *Journal of synchrotron radiation* **2012**, *19*, 647-653.

[11] M. A. Reus, L. K. Reb, D. P. Kosbahn, S. V. Roth, P. Müller-Buschbaum, *Journal of applied crystallography* **2024**, *57*.

[12] D. Nečas, P. Klapetek, *Open Physics* **2012**, *10*, 181-188. https://doi.org/doi:10.2478/s11534-011-0096-2.

[13] J. A. Steele, E. Solano, D. Hardy, D. Dayton, D. Ladd, K. White, P. Chen, J. Hou, H. Huang, R. A. Saha, *Advanced Energy Materials* **2023**, *13*, 2300760.

[14] M. A. Reus, L. K. Reb, A. F. Weinzierl, C. L. Weindl, R. Guo, T. Xiao, M. Schwartzkopf, A. Chumakov, S. V. Roth, P. Müller‐Buschbaum, *Advanced Optical Materials* **2022**, *10*, 2102722.

[15] K. Shirzad, C. Viney, *Journal of the Royal Society Interface* **2023**, *20*, 20230242.

[16] V. Savikhin, H.-G. Steinrück, R.-Z. Liang, B. A. Collins, S. D. Oosterhout, P. M. Beaujuge, M. F. Toney, *Applied Crystallography* **2020**, *53*, 1108-1129.

[17] B. L. Henke, E. M. Gullikson, J. C. Davis, *Atomic data and nuclear data tables* **1993**, *54*, 181-342.

[18] F. C. Hanusch, E. Wiesenmayer, E. Mankel, A. Binek, P. Angloher, C. Fraunhofer, N. Giesbrecht, J. M. Feckl, W. Jaegermann, D. Johrendt, *The journal of physical chemistry letters* **2014**, *5*, 2791-2795.

[19] J. I. Pankove, *Optical processes in semiconductors*, Courier Corporation, **1975**.

[20] J. Mooney, P. Kambhampati, *Vol. 4*, ACS Publications, 2013, pp. 3316-3318.

[21] G. Pan, S. Yin, L. F. Huber, Z. Li, T. Tian, L. V. Spanier, H. Zhong, T. Guan, C. R. Ehgartner, N. Hüsing, *Small* **2025**, 2409856.

[22] P. Müller-Buschbaum, *Analytical and bioanalytical chemistry* **2003**, *376*, 3-10.
